# Posterior segment inflammatory outcomes assessed using fluorescein angiography in the STOP-UVEITIS study

**DOI:** 10.1186/s40942-020-00245-w

**Published:** 2020-10-06

**Authors:** Mohammad Ali Sadiq, Muhammad Hassan, Rubbia Afridi, Muhammad Sohail Halim, Diana V. Do, Yasir J. Sepah, Quan Dong Nguyen, for the STOP-UVEITIS Investigators

**Affiliations:** 1 Department of Ophthalmology, University of Louisville, Louisville, KY USA; 2 Spencer Center for Vision Research, Byers Eye Institute, Stanford University, 2370 Watson Court, Suite 200, Palo Alto, CA 94303 USA

**Keywords:** Uveitis, Non-infectious, IL-6, Tocilizumab, Fluorescein angiography, Clinical trial

## Abstract

**Background:**

Although fluorescein angiography (FA) is a frequently used imaging modality in patients with non-infectious uveitis (NIU), it has not been reliably used for objective assessment of posterior segment inflammatory outcomes in these patients. In this index study we report the posterior segment inflammatory outcomes of two different doses of intravenous (IV) infusions of tocilizumab (TCZ), an IL-6 inhibitor, in eyes with NIU using a semi-quantitative FA scoring system.

**Methods:**

STOP-Uveitis is a randomized, multi-center clinical trial conducted at 5 clinical centers across the United States. The study evaluated the role of TCZ in patients with NIU. Thirty-seven (37) patients with NIU were randomized into one of two treatment groups in a ratio of 1:1. Group 1 received IV infusions of 4 mg/kg TCZ and group 2 received IV infusions of 8 mg/kg TCZ. Infusions were given every 4 weeks in both groups until month 6 (primary endpoint). Posterior segment inflammatory outcomes were assessed by evaluating FA at baseline and month 6 by graders at a central reading center. A previously reported, semi-quantitative, scoring system for FA was used to assess signs that represent ongoing inflammatory processes in the posterior segment. These signs included optic disc hyperfluorescence, macular edema, retinal vascular staining and/or leakage, capillary leakage, retinal capillary nonperfusion, neovascularization of the optic disc, neovascularization elsewhere, pinpoint leaks, and retinal staining and/or subretinal pooling. Statistical significance was set at p < 0.05. Main outcome measures included change in posterior segment inflammation as assessed using FA at month 6.

**Results:**

37 eyes (37 patients) were randomized in the STOP-Uveitis study. 30 eyes were found to be eligible for this sub-study based on study criteria. Seven eyes had ungradable images at either baseline or month 6 and were therefore excluded from the analysis. The reduction in FA inflammatory scores at month 6 were statistically significant in both groups (p < 0.05). The difference between the two groups was not significant (p = 0.351).

**Conclusions:**

IV infusions of tocilizumab (both 4 and 8 mg/kg) are effective in improving posterior segment inflammation in eyes with NIU. A semi-quantitative FA scoring system may be used as a reliable outcome measure for assessment of posterior segment inflammation.

ClinicalTrials.gov Identifier: NCT01717170

## Background

Uveitis and its complications, account for approximately 5–20% of cases of preventable blindness in the developed world, and up to 25% of cases in developing countries [1]. The primary aim in the management of patients with uveitis is to achieve and maintain quiescence in inflammation and prevent the development of ocular adverse events. Although corticosteroids are still considered the first-line therapy for patients with non-infectious uveitis (NIU), they are associated with considerable local and systemic side effects. Therefore, steroid-sparing agents such as Tocilizumab (TCZ), are currently under investigation. TCZ is a recombinant humanized monoclonal antibody directed against IL-6; which is a pro-inflammatory cytokine with a proven role in immune mediated diseases such as uveitis. The **S**tudy of the safety, tolerability, and bioactivity of **T**ocilizumab **O**n **P**atients with non-infectious Uveitis (STOP-Uveitis) study was the first multicenter, randomized, open label clinical trial evaluating two different doses of TCZ (4 mg/kg and 8 mg/kg) in patients with NIU. The study has previously reported a favorable safety and efficacy profile at the month 6 primary endpoint of the study [2].

Although the vitreous haze (VH) score is a commonly used clinical marker for posterior segment inflammation in patients with NIU, it may not be the most reliable marker since every patient may not initially present with significant vitreous haze. Therefore, alternative methods to reliably assess posterior segment inflammatory outcomes in patients with uveitis need to be explored. Fluorescein Angiography (FA) is a frequently used imaging modality in patients with NIU. However, FA analyses are qualitative, and grader dependent, making comparisons among different end points challenging. To overcome this challenge, different fluorescein angiography scoring schemes have been previously reported [3–5]. The Angiography Scoring for Uveitis Working Group (ASUWOG) have reported a semi-quantitative scoring system using fluorescein and indocyanine angiography, to assess posterior segment inflammation in patients with uveitis [6]. We used this particular scoring system for the grading of posterior segment inflammation in our study because it evaluates both the extent (area of fundus involved) and the magnitude (severity) of the most important inflammatory parameters in patients with NIU including; optic disc hyperfluorescence; macular edema; retinal vascular staining/leakage; capillary leakage; retinal capillary nonperfusion; neovascularization of the optic disc (NVD); neovascularization elsewhere (NVE); pinpoint leaks; retinal staining/pooling. (Table 1) In addition, this scoring system has been previously utilized extensively in clinical studies evaluating several ocular inflammatory diseases with the authors reporting good inter-grader agreement [7–12].

**Table 1:** Maximum scores for individual inflammatory signs in the Angiography Scoring for Uveitis WOrking Group (ASUWOG) fluorescein angiography scoring system

Angiographic sign	Maximum score
Optic disc hyperfluorescence	3
Macular edema	4
Retinal vascular staining/leakage	7
Capillary leakage	10
Retinal capillary nonperfusion	6
Neovascularization of the optic disc (NVD)	2
Neovascularization elsewhere (NVE)	2
Pinpoint leaks	2
Retinal staining/pooling	4
Total	40

In this index study, we assessed posterior segment inflammatory outcomes using a FA scoring system in patients with NIU treated with two different monthly infusions of Tocilizumab.

## Methods

Data from the STOP-Uveitis study was utilized for this study. The STOP-Uveitis study was a multicenter, randomized, open-label clinical trial designed to assess the safety and efficacy of repeated intravenous (IV) infusions of two doses of TCZ (4 mg/kg and 8 mg/kg) in subjects with non-infectious uveitis (NIU). The infusions were administered over one hour.

The STOP-Uveitis trial is registered at http://www.clinicaltrials.gov under the identifier NCT01717170 and was conducted in compliance with the United States Code of Federal Regulations Title 21, the Declaration of Helsinki, and the Harmonized Tripartite Guidelines for Good Clinical Practice (1996). The study was approved by local institutional review boards for selected sites and by a central review board for others. Signed informed consents were obtained from all the participants in the study.

Fluorescein angiography images were acquired using the Zeiss FF-450 Fundus Camera (Carl Zeiss Meditec Inc., Dublin, CA) at 2 study sites, and using the Topcon TRC-50DX (Topcon Corporation, Tokyo, Japan) fundus camera at 3 study sites. OCT imaging was performed using the Heidelberg Spectralis HRA + OCT device (Heidelberg Engineering, Heidelberg, Germany) at all the study sites.

### Patient eligibility and exclusion criteria

Patients were included in the index sub-study analysis if they met the following criteria: (1) participation in the STOP-Uveitis clinical trial and completion of the primary end-point visit of month 6; (2) availability of gradable fluorescein angiography images at baseline and month 6. Eyes with ungradable images at the baseline and/or month 6 visit were excluded from the analysis. Inclusion and exclusion criteria of the STOP-Uveitis study have been published previously [2].

### Randomization and intervention

During the treatment period (Day 0 to month 5), patients in Group 1 (4 mg/kg, low-dose group) and Group 2 (8 mg/kg, high-dose group) received intravenous (IV) infusions of TCZ on days 0, 30, 60, 90, 120 and 150. During the follow-up period (month 6 to 12), treatment was optional and administered only if patients qualified for treatment based on re-treatment criteria.

### Fluorescein angiography analysis for posterior segment inflammatory outcomes

**Fig. 1:**
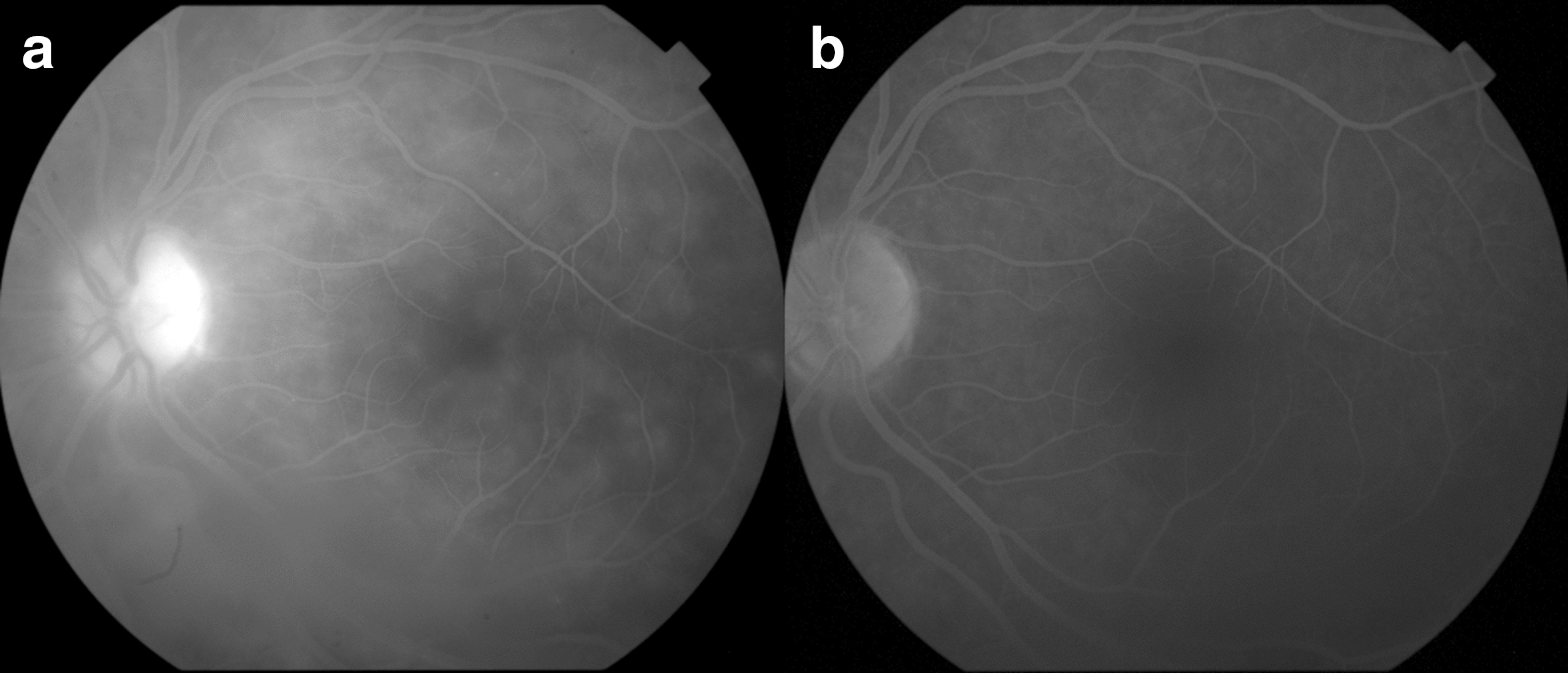
Representative images from a study patient at: **a** Baseline, showing diffuse areas of capillary leakage and an area of disc hyperfluorescence (inflammatory score 8) and at; **b** Month 6, showing a significant improvement in capillary leakage and disc hyperfluorescence (inflammatory score 1)

The ASUWOG fluorescein angiography scoring system was used to determine the inflammatory scores in the study eyes at baseline and Month 6. This scoring system assigns scores to various inflammatory signs on FA with a maximum total score of 40 (Table 1). Two independent certified graders assessed the images at a central reading center, the Ocular Imaging Research and Reading Center (OIRRC, Sunnyvale, CA). The graders were masked to both the timepoint and treatment when scoring images. A senior grader adjudicated any discrepancies between the two graders. Figure 1 provides representative FA images of a patient at the baseline and month 6 visit.

### Outcome measures

Mean change in posterior segment inflammation as assessed using the FA inflammatory score, from baseline to month 6, in the two study groups.

### Statistical analysis

As the STOP-Uveitis study was an exploratory, pilot phase 1/2 study, the sample size was not chosen based on statistical calculation. The sample size per dose group was chosen based on clinical judgment; it was estimated that approximately 18 subjects in each group may be able to provide initial information regarding the safety and efficacy of TCZ infusions in patients with uveitis. Non-parametric tests were used to analyze the data. Quantitative data was analyzed for significance within groups using Wilcoxon Signed Rank test and between groups using Mann Whitney U test. Stata V14.1 (Stata Corp, TX) was used for all statistical analyses. Statistical significance was set at p < 0.05.

## Results

A total of 37 patients were enrolled in the STOP-Uveitis study. Baseline demographic characteristics of the two study groups have been reported previously and are shown in Table 2. Out of 37 patients, 30 patients (30 eyes) were included in this sub-study analysis based on the inclusion and exclusion criteria. Seven subjects (7 eyes) had ungradable FA images and were therefore excluded from the study.

**Table 2:** Demographic characteristics and baseline details of the study participants

Study group	Group 1 (4 mg/kg) n = 18	Group 2 (8 mg/kg) n = 19	P-value
Gender [% female (n)]	61.1 (11)	57.9 (11)	1.0
Race [% white (n)]	88.9 (16)	79.0 (15)	0.11
Race [%Asian (n)]	0.0 (0)	10.5 (2)	
Race [% Hispanic or Latin (n)]	11.1 (2)	0.0 (0)	
Race [% African American (n)]	0.0 (0)	10.5 (2)	
Age (mean years ± SD)	43 ± 16.52 (18 – 68)	41 ± 18.0 (19 – 85)	0.79
Visual acuity (mean ± SD)	34.28 ± 15.39	41.11 ± 14.98	0.18
Vitreous haze (mean ± SD)	1.25 ± 0.82	1.5 ± 0.95	0.48
Central macular thickness (mean ± SD)	411.97 ± 215.43	308.76 ± 91.03	0.08
Lens status (% pseudophakic)	27.8 (5)	15.8 (3)	0.45
Treatment Naïve (%)	55.5 (10)	63.1 (12)	0.74
Systemic corticosteroid use (%(n))	27.8 (5)	21.1 (3)	0.45
Corticosteroid dose (mean ± SD)	18.0 ± 12.5	20.0 ± 14.1	0.86
Prior IMT use (%(n))	22.2 (4)	26.3 (5)	1.0
Disease Type [% (n)]			
Intermediate	16.7 (3)	15.8 (3)	0.30
Posterior	22.2 (4)	5.3 (1)	
Panuveitis	61.1 (11)	78.9 (15)	
Disease category [% (n)]			
Active uveitis with no treatment	55.6 (10)	68.4 (13)	0.51
Active uveitis with treatment	44.4 (8)	31.6 (6)	
Underlying disease [% (n)]			
Sarcoidosis	5.6 (1)	5.3 (1)	0.27
Vogt-Koyangi-Harada Syndrome		10.5 (2)	
Birdshot choroidopathy	11.1 (2)		
Punctate inner choroiditis	5.6 (1)		
Bechet disease	5.6 (1)		
Tubulointerstitial nephritis and uveitis (TINU syndrome)	5.6 (1)		
Idiopathic	66.7 (12)	84.2 (16)	

### Fluorescein angiography inflammatory outcomes

Table 3 shows the median changes in the FA inflammatory scores from baseline to month 6 in the study groups. At month 6, the overall (groups combined) median FA inflammatory score improved from 6.00 ± 3.26 (median ± SD/standard deviation) at baseline to 2.00 ± 3.40 at month 6 (p = 0.0006). The median FA inflammatory score in group 1 decreased from 7.50 ± 3.56 at baseline to 2.00 ± 4.05 at month 6 (p = 0.019). Similarly, the median FA inflammatory score decreased from 5.50 ± 2.67 at baseline to 2.00 ± 2.50 at month 6 in group 2 (p = 0.013). The difference in median improvement of FA inflammatory score between the two study groups was not statistically significant (p > 0.05).

**Table 3:** Median fluorescein angiography inflammatory scores at baseline and month 6

	Baseline	Month 6	p-value
Combined, n = 30	6.00 ± 3.26	2.00 ± 3.40	0.0006
Group 1 (4 mg/kg), n = 15	7.50 ± 3.56	2.00 ± 4.05	0.019
Group 2 (8 mg/kg), n = 15	5.50 ± 2.67	2.00 ± 2.50	0.013

All 30 patients analyzed in the study had an inflammatory score greater than 0 at baseline, suggesting some degree of posterior segment inflammation at baseline. Table 4 demonstrates the percentage of patients in each group that showed improvement, worsening or no change in FA inflammatory scores at month 6. 25 patients (83.3%) showed an improvement, three patients (10%) showed worsening and two (6.6%) showed no change in the inflammatory score at month 6. There was no significant difference in the percentage of patients that showed improvement in the inflammatory scores at month 6 between the two study groups. All three patients that showed worsening in the inflammatory score did so due to a 1-point increase in capillary leakage at the month 6 visit.

**Table 4:** Percentage of patients showing improvement, worsening or no change in the inflammatory score at month 6

	Improvement [% (n)]	Worsening [% (n)]	No change [% (n)]
Combined, n = 30	83.3 (25)	10 (3)	6.6 (2)
Group 1 (4 mg/kg), n = 15	87.5 (14)	12.5 (2)	0
Group 2 (8 mg/kg), n = 15	78.5 (11)	7.1 (1)	14.2 (2)

## Discussion

There are several therapeutic agents that are currently being investigated for NIU. However, adalimumab is the only current food and drug administration (FDA) approved non-corticosteroid therapy for adults with non-infectious uveitis. Tocilizumab (TCZ) is a novel recombinant monoclonal antibody directed against IL-6 receptors. It is currently used as an off-label therapy for patients with refractory uveitis and ocular inflammatory diseases. We have previously reported favorable outcomes of patients with NIU treated with TCZ in the STOP-Uveitis study [2, 13]. These included a statistically significant improvement in BCVA, CRT and vitreous haze.

The standardization of uveitis nomenclature vitreous haze score (SUN-VH) is currently the most frequently used parameter for grading of posterior segment inflammation in clinical trials. It relies on the subjective assessment of fuzziness of the posterior segment as assessed using indirect ophthalmoscopy. However, given the significant variability of patients with NIU, many patients may present with significant visual morbidity without the accompanying vitreous haze [14, 15]. In addition, a significant number of patients may not present with a vitreous haze score high enough to demonstrate a 2-step improvement during the study. It is also often challenging to recruit patients with at least 2 + vitreous haze. In the STOP-Uveitis study, patients were defined as having active uveitis and were included in the study if they had vitreous haze greater than or equal to 1 at baseline. A 2-step improvement in the VH score was achieved by 32.3% (10 out of 37) subjects in the STOP-Uveitis study. Only 23 out of 37 subjects initially enrolled had the potential for a 2-step improvement in VH, which suggested that the actual 2-step improvement in patients that had the potential for 2-step improvement in VH was 43.3%. Therefore, assessment of disease activity and progression in patients with NIU cannot be reliably assessed using VH alone.

To overcome this challenge, we used a semi-quantitative FA scoring system to report the posterior segment inflammatory outcomes in these patients. Fluorescein angiography (FA) is a commonly used imaging modality in patients with posterior segment disease. However, given the qualitative mode of assessment and variability in grader assessment, it has not been frequently used to report large-scale data in patients with NIU. Therefore, the role of FA as a reliable disease marker in clinical trials has not realized its fullest potential. We used a novel FA scoring system proposed by the ASUWOG group, which is a semi-quantitative scoring system that was initially proposed in 2008, to evaluate both the severity and extent of posterior segment inflammation in patients with NIU. In this index study, we report improved posterior segment inflammatory outcomes in patients with NIU treated with TCZ at the month 6 visit. Both the low-dose and high-dose groups showed a significant reduction in inflammatory scores.

A reliable scoring system in patients with uveitis has several advantages: it can help support the diagnosis; reduce personal bias, improve assessment during follow-up visits and communication between physicians, and allow an objective way of analyzing data for clinical trials. The FA scoring system reported by the ASUWOG group has been utilized in multiple studies previously [6, 10]. Mantovani et al. [12] studied the inter-observer variability in the ASUWOG scoring system. The study concluded that the ASUWOG scoring system is a reliable method for assessing degree of ocular inflammation. Kang et al. [16] also utilized the ASUWOG FA scoring system to study patients with Adamantiades-Bechet’s disease patients for long-term progression of retinal vasculitis, an essential feature of the disease. Using this scoring system, they were able to show that there is no significant difference in the degree of inflammation between the first episode and subsequent flares in the patients. Furthermore, they also demonstrated that there is a significant difference in inflammatory signs between the active and quiescent phases of the disease.

All 30 patients analyzed in this study had a baseline inflammatory score greater than 0, suggesting some degree of inflammation at baseline. 83% of these patients showed improvement in the inflammatory scores at month 6. Two patients demonstrated no change in the inflammatory scores and three patients showed worsening. All three patients that showed worsening had a 1-point increase in the inflammatory scores attributed to mild worsening of macular leakage at month 6. None of these three patients that showed worsening in the inflammatory scores demonstrated a visual decline. This finding reiterates the importance of using the FA inflammatory score in combination with other clinical parameters to make accurate assessments regarding disease management.

We assessed the correlation between the changes in the FA inflammatory score, BCVA and CRT during the study and found only a weak correlation between these study variables. This finding re-emphasizes again the importance of analyzing all outcome measures in combination. Non-infectious uveitis is a group of diseases that often shows great variability in disease presentation and the underlying pathological processes involved may be significantly different. Assessment using FA will only add to the armamentarium at our disposal for accurate assessment and follow up of patients with NIU, and caution must be exercised when using it in isolation for management decisions. A composite scoring system utilizing all modes of assessment including BCVA, CRT, FA inflammatory score, and VH for example, may be more helpful in improving the accuracy and reliability of assessments in these patients.

The strengths of our study include the mandatory monthly treatment design and well-characterized study population of a multicenter clinical trial. Previous studies of TCZ have largely been limited to case reports and short case-series. All images were graded at a central reading center in a controlled environment. In addition, we used a previously verified semi-quantitative scoring system for assessment of posterior segment inflammatory outcomes in our study.

The mean FA inflammatory score at baseline was 6.00 ± 3.26, suggesting a low-grade of posterior segment inflammation at baseline. This may either suggest a low sensitivity of the scoring system for detecting posterior segment disease or more likely that the patients did not have significant posterior segment inflammatory findings at baseline. In the STOP-Uveitis study patients with active uveitis were enrolled in the study. Active uveitis was defined as the presence of ≥ 1 vitreous haze at baseline. The presence of other posterior segment inflammatory findings was therefore not necessary at baseline, which may explain the relatively low baseline FA inflammatory score.

Possible limitations of our study include the relatively small sample size, post-hoc nature of analysis, and the short duration of follow-up. Data from seven patients in the study (18.9%) could not be utilized for this sub study analysis due to the presence of ungradable images at either baseline or at month 6. In addition, the study was not statistically powered to compare the efficacy of the two doses and to assess the efficacy among the different causes of uveitis in the study.

## Conclusions

Our study reports that monthly infusions of tocilizumab significantly improve posterior segment inflammatory outcomes in patients with non-infectious uveitis. Fluorescein angiography analyses using an objective scoring system may be employed for assessment of posterior segment inflammation in these patients. We propose employing such scoring methods in combination with other outcome measures, to help uveitis specialists make reliable management decisions in patients with non-infectious uveitis.

## Data Availability

The datasets used and/or analysed during the current study are available from the corresponding author on reasonable request.

## Abbreviations

ASUWOG: Angiography Scoring for Uveitis WOrking Group
BCVA: Best corrected visual acuity
CRT: Central retinal thickness
FA: Fluorescein angiography
IV: Intravenous
NIU: Non-infectious uveitis
SUN: Standardization of uveitis nomenclature
TCZ: Tocilizumab
VH: Vitreous haze
